# Enhanced recovery after surgery (ERAS) in elective intertrochanteric fracture patients result in reduced length of hospital stay (LOS) without compromising functional outcome

**DOI:** 10.1186/s13018-019-1238-2

**Published:** 2019-07-09

**Authors:** Yan Kang, Jianxing Liu, Haihong Chen, Wang Ding, Jianqing Chen, Bin Zhao, Xiaofan Yin

**Affiliations:** 1 Orthopaedic Department, Minhang Center Hospital, Fudan University, 170 Xin Song Road, Shanghai, People’s Republic of China; 2 Orthopaedic Department, Minhang Center Hospital, Fudan University, 170 Xin Song Road, Shanghai, People’s Republic of China; 3 Orthopaedic Department, Minhang Center Hospital, Fudan University, 170 Xin Song Road, Shanghai, People’s Republic of China; 4 Orthopaedic Department, Minhang Center Hospital, Fudan University, 170 Xin Song Road, Shanghai, People’s Republic of China; 5 Orthopaedic Department, Minhang Center Hospital, Fudan University, 170 Xin Song Road, Shanghai, People’s Republic of China; 6 Orthopaedic Department, Minhang Center Hospital, Fudan University, 170 Xin Song Road, Shanghai, People’s Republic of China; 7 Orthopaedic Department, Minhang Center Hospital, Fudan University, 170 Xin Song Road, Shanghai, People’s Republic of China

**Keywords:** Intertrochanteric fracture, Enhanced recovery after surgery (ERAS), Length of stay (LOS), Intramedullary fixation, Proximal femoral nail anti-rotation (PFNA)

## Abstract

**Background:**

Enhanced recovery after surgery (ERAS) has rapidly gained popularity among hip or knee arthroplasty area which can decrease hospital length of stay (LOS). However, limited data exist regarding its safety and efficacy among intertrochanteric fracture patients. The purpose of this study was to determine if LOS associated with intertrochanteric fracture patients can be improved following an existing orthopedic ERAS procedure.

**Methods:**

We reviewed the outcomes of all patients who had been treated with the PFNA intramedullary fixation at our institution. Open fractures, metastatic pathological fractures, patients unable to walk independently before fracture and patients with Alzheimer's disease were excluded. A quasi-experimental study was adopted between patients treated in an ERAS after intramedullary fixation with those rehabilitated on a traditional pathway. Clinical and demographic data were collected among the two pathway cohorts including LOS, Harris hip scores (HHS), visual analog scale (VAS), and activity of daily living scale (ADL).

**Results:**

A total of 100 intertrochanteric fracture patients (ERAS pathway 50 cases, traditional care pathway 50 cases) were selected between January 2016 and December 2017 met the inclusion criteria. ERAS procedure was associated with shorter LOS, lower postoperative VAS scores, reduced opioid consumption, earlier mobilization, significant improvement in the mean HHS scores at 3 months postoperatively, lower risk of complications, lower rates of readmission, and reoperation and higher likelihood of being discharged home. The mean LOS decreased from 8.21 ± 0.83 days to 5.82 ± 0.64 days after implementation of the evidence-based orthopedic ERAS pathway (*p* < 0.05).

**Conclusions:**

This series of intertrochanteric fracture patients treated with the orthopedic ERAS procedure demonstrated that the procedure is capable of reducing LOS and preserving hip function without compromising functional outcome. This improvement was possible without a concomitant increase in postoperative complications and readmission rates.

**Level of Evidence:**

Therapeutic Level IV. See Instructions for Authors for a complete description of levels of evidence.

## Introduction

Intertrochanteric fracture is a major public health issue which has a high mortality rate within one year [1–4]. Intramedullary fixation system has proven to be highly clinically effective interventions, with high rates of success in terms of reduced pain, improved quality of life, and hip function at medium-term and long-term follow-up [1–4]. More recently, AO/ASIF (Association for the Study of Internal Fixation) designed a new internal fixation system PFNA (proximal femoral nail anti-rotation, PFNA; Synthes, Oberdorf, Switzerland), which is characterized by maintaining AO strong fixation and biomechanical stability [5–8]. Additionally, consistent with the essence of BO theory and minimally invasive surgery concept, it is more suitable for all types of intertrochanteric fractures [9, 10].

LOS associated with intertrochanteric fracture is a major public health issue due to the aging population [11–14]. Some investors predicted that any further decreases in the LOS would correspond with increases in postoperative complications [5]. To date, no research has shown that decreases in LOS after treatment of intertrochanteric fracture can be achieved in combination with the reduction of postoperative complications, readmissions, and therefore mortality [15–17].

The concept of ERAS originated from colorectal surgery in the late 20th century which have successfully demonstrated benefits to the surgical patient in colorectal and upper gastrointestinal [11, 12]. Previous studies have reported that clinical application of ERAS in the artificial joint replacement domain may reduce postoperative LOS and mortality, with increased satisfaction and safety after discharge [18–21]. However, the relative merits and drawbacks of this concept are not well described in intertrochanteric fracture patients.

In summary, the potential of ERAS for patients undergoing intramedullary fixation is great, but the overall level of evidence is low. The purpose of this study was to evaluate the effect of an evidence-based ERAS clinical pathway on perioperative outcomes in intertrochanteric fracture patients undergoing intramedullary fixation.

## Materials and methods

### Study design

**Fig. 1:**
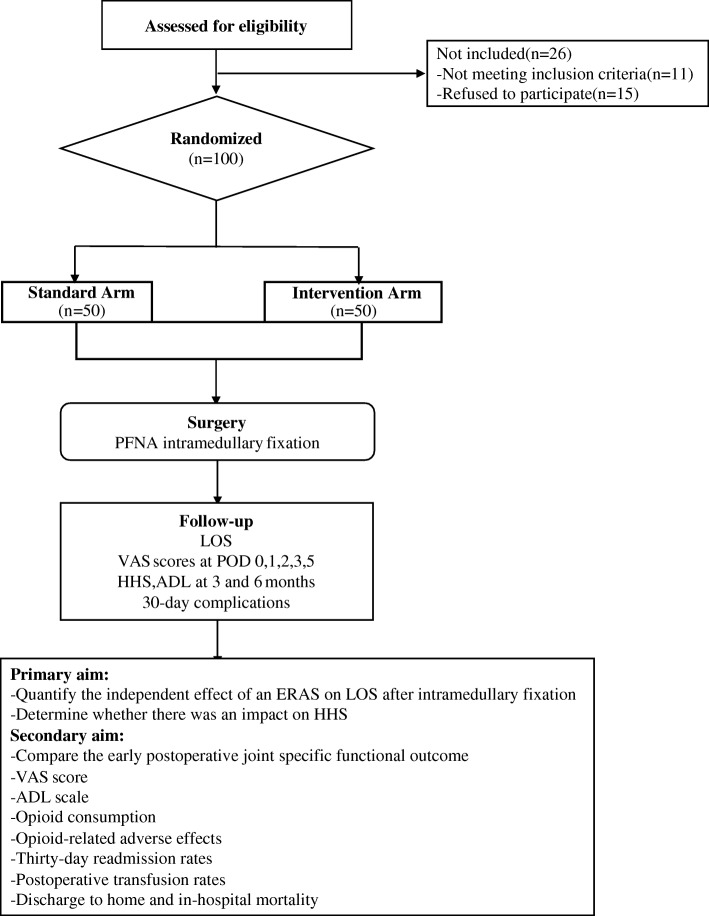
Flow chart of patients in the randomized clinical trial

This was a quasi-experimental design study that compared a prospective cohort (ERAS: January–December 2017) with a historical standardized care pathway cohort (control: January–December 2016). Emergency admissions with a primary diagnosis of intertrochanteric fracture between January 2016 and December 2017 in our hospital were selectively enrolled. Ethical approval was obtained from the internal review board, and orthopedic departmental approval was gained to proceed with prospective data collection. All patients were treated with PFNA intramedullary fixation at our institution. Inclusion criteria consisted of patients who had undergone surgery with a standardized care pathway and those who had undergone surgery with the ERAS pathway. Radiological data were reviewed to identify the fractures type based on the AO classification. Patients undergoing PFNA intramedullary fixation surgery were eligible to participate in the prospective arm of the study. Written and verbal informed consent was obtained from each patient before inclusion in the study. Exclusion criteria comprised patients with open fractures, metastatic pathological fractures, patients unable to walk independently before fracture, and inability to follow verbal or written instructions (Fig. 1).

All patients received their operation at our hospital where elective surgeries take place predominantly. Here, ERAS programs for arthroplasty and preoperative multidisciplinary collaboration had been already established. For each of the study periods (control and ERAS), patients were enrolled consecutively. The consecutive data collected for both groups were compared for age, gender, body mass index (BMI), LOS, the pre-, and postoperative HHS. Preoperative comorbid status was assessed by means of the American Society of Anesthesiologists (ASA) score and the presence of diabetes mellitus, hypertension, hypercholesterolemia, chronic obstructive pulmonary disease (COPD), ischemic heart disease (IHD), and atrial fibrillation (AF) [22]. Thirty-day were also recorded, including readmission rates.

The primary outcome measure was LOS at discharge, and the secondary outcome measure was the patient’s early postoperative joint specific function (HHS score). The other secondary outcomes included VAS score, ADL scale, opioid consumption, opioid-related adverse effects, 30-day readmission rates, postoperative transfusion rates, discharge to home, and in-hospital mortality. The other secondary outcomes included VAS score, ADL scale, opioid consumption, opioid-related adverse effects, 30-day readmission rates, postoperative transfusion rates, discharge to home, and in-hospital mortality.

### Orthopedic ERAS pathway

The basic components of the multi-disciplinary and multimodal ERAS pathway which we used are shown in Table 1. The principles include a preoperative educational program, opioid-free anesthesia and postoperative period, promotion of early independence, and supported and safe discharge. The patients were prescribed 400 mg of oral Celecoxib 1 h prior to surgery and reduced to 200 mg for elderly patients (> 70 years) or those with a low BMI (< 25 kg/m^2^) on the day of surgery. Opioid-free spinal anesthesia was preferred at the discretion of the anesthetist. Intravenous (IV) tranexamic acid (1 g) was given preoperatively if not contra-indicated. Dexamethasone 8 mg (IV) and 40 mg of parecoxib sodium (IV) were given intraoperatively. Early mobilization was facilitated under a rehabilitation therapist. Oral postoperative opioid-sparing analgesia including non-steroidal anti-inflammatory drugs (celecoxib 200 mg, two times a day) provided on a regular basis (assuming no contraindications) and tramadol (100 mg to 200 mg up to two times a day, when necessary) were routinely prescribed when the patient returned to the ward.

**Table 1:** Enhanced recovery after orthopedic surgery pathway (ERAS)

Preoperative	
Pre-operative educational program	
Oral multimodal analgesia (celebrex)	
Opioid-free anesthesia: short-acting spinal preferred (mepivacaine)	
Intraoperative	
Intravenous dexamethasone	
2 L of lactated Ringer’s	
Tranexamic acid	
Postoperative	
Early mobilization: physical therapy session on the day of surgery	
Opioids avoiding: scheduled NSAIDs prn	
Nausea and vomiting control	
Supported discharge	

### Statistical analysis

Descriptive statistics were calculated using Statistical Package for Social Sciences (SPSS) (Version 24.0, IBM, Armonk, NY, USA). Demographic and baseline clinical characteristics were compared using two-tailed unpaired Student’s *t* test or chi-square test as appropriate. Mean values for normally distributed continuous data such as LOS (nights in hospital), HHS scores, and complication rates between the two groups were compared using two-tailed unpaired Student’s *t* tests or chi-squared test as appropriate. The mean and standard deviation, alongside the minimum and maximum values, were determined for all outcomes. A *p* value < 0.05 was considered statistically significant.

## Results

A total of 100 intertrochanteric fracture patients (ERAS pathway: 50 cases, Traditional care pathway: 50 cases) were selected between January 2016 and December 2017 while following the ERAS and traditional care pathway. The average age of the intertrochanteric fracture patients in our study have no statistical differences in the ERAS and traditional pathway groups, respectively, and 68% were female. All of the patients underwent PFNA intramedullary fixation. There were no baseline statistical differences between groups in age, gender, BMI, ASA scores (Table 2). Spinal anesthesia was adopted as the primary anesthetic between ERAS group and the traditional rehabilitation group.

**Table 2:** The characteristics of the patients in each group

	Traditional (*n* = 50)	ERAS (*n* = 50)	*p* value	
Age (years)	78.32 ± 8.24	77.81 ± 8.14	0.528	
Gender (M:F)	16:34	15:35	0.232	
Operative side (left: right)	28:22	27:23	0.418	
BMI	27.5	28	0.311	
Pre-operative Hb	10.5	10.8	0.134	
ASA grade (II/III, cases)	41:9	40:10	0.146	
Pre-operative HHS	48.3	49.3	0.15	
Mean operating time (min)	41.82 ± 3.53	42.34±3.21	0.162	
Mean volume of blood loss (ml)	137.54 ± 20.22	141.23 ± 22.31	0.231	

*BMI* body mass index, *ASA* American Society of Anesthesiologists, *HHS* Harris hip score

The primary outcome of this investigation was hospital LOS. The median LOS in the ERAS group was 5~7 days (average, 5.82 ± 0.64 days) compared to 7~9 days (average, 8.21 ± 0.83 days) in the traditional rehabilitation group (Table 2). The difference in mean LOS between the two groups was statistically significant. Furthermore, significantly more patients in the ERAS group left the hospital within 4 days of the operation.

The clinical outcomes in both groups are shown in Table 3. The preoperative VAS scores of the two groups have no statistical difference. The postoperative VAS scores of the two groups were compared as shown in Table 3. Patients in the ERAS pathway had significantly lower VAS scores on postoperative day (POD 0) and the subsequent POD 1, POD 2, and POD 3 compared to the traditional pathway cohort. There was no significant difference in VAS score between the two groups on POD 5. Opioid consumption was similar on POD 0, but significantly reduced for the ERAS pathway cohort on POD 1, POD 2, POD 3, and POD 5. In turn, these patients had significantly less treatment for opioid-induced adverse events.

**Table 3:** Mean LOS, VAS scores, mean HHS, and mean ADL scores

	Traditional (*n* = 50)	ERAS (*n* = 50)	*p* value
Mean LOS (days)	8.21 ± 0.83	5.82 ± 0.64	0.028
Mean VAS scores preoperative	8.33 ± 1.41	8.36 ± 1.35	0.461
POD 0	7.33 ± 1.41	6.63 ± 1.61	< 0.001
POD 1	6.51 ± 1.52	5.82 ± 1.42	< 0.001
POD 2	5.72 ± 1.91	4.31 ± 2.14	< 0.001
POD 3	4.53 ± 1.61	3.52 ± 1.81	< 0.001
POD 5	2.61 ± 1.94	2.44 ± 1.72	0.265
Mean HHS at 3 months	80.51 ± 5.93	84.72 ± 6.14	0.016
Mean HHS at 6 months	90.32 ± 6.82	90.51 ± 6.51	0.542
Mean ADL at 3 months	82.61 ± 3.23	83.31 ± 3.51	0.387
Mean ADL at 6 months	89.44 ± 4.11	89.14 ± 3.92	0.153

*POD* postoperative day, *HHS* Harris hip score, *ADL* activities of daily living

Early mobilization (< 24 h) was achieved in all of the patients in the ERAS group. Eighty-two of those patients mobilizing early were successfully discharged within 4 days or less. This is in contrast to the control group, which demonstrated only 48% of patients mobilizing within 24 h of their operation. There was a significant improvement in the mean HHS scores in ERAS group at 3 months but no statistically significant differences between the groups at 6 months postoperatively. Besides, there were no statistical differences in the mean ADL scores both at 3 and 6 months postoperatively, see Table 3. In conclusion, we considered evidence-based ERAS clinical pathway as a positive procedure for intertrochanteric fracture patients especially in the perioperative period.

Summary of postoperative complications in 30 days is listed in Table 4 for both groups. There were statistically significant differences in complications between ERAS and control group. All the wounds healed primarily without clinical complications such as infection, deep venous thrombosis of lower extremity, and pulmonary embolism during the follow-up period. There were 4 cases of complications in the ERAS group and 10 cases in the traditional rehabilitation group postoperative, which have significant statistically difference (Table 4). No deaths occurred in either group (30-day follow-up).

**Table 4:** Comparison of 30-day complications between the two groups

	Traditional (*n* = 50)	ERAS (*n* = 50)	*p* value
Complications	11	4	< 0.001
GI bleeding	1	0	
MI	1	0	
Symptomatic DVT/PE	0	0	
Re-admission	0	0	
Wound infection	0	0	
Deep infection	0	0	
CVA	2	1	
UTI	2	1	
RTI	2	1	
Delirium	3	1	

*DVT* deep venous thrombosis *UTI* urinary tract infection, *RTI* respiratory tract infection, *PE* pulmonary embolism, *CVA* cerebral vascular accident, *GI* gastrointestinal, *RTI* respiratory tract infection, *MI* myocardial infarction

## Discussion

This study shows that LOS after intramedullary fixation in the intertrochanteric fracture can be significantly reduced by using ERAS pathway, while maintaining better early functional outcomes to those rehabilitated in a traditional manner, and without increasing the rate of complications or mortality postoperatively.

Intertrochanteric fractures commonly occurred in the elderly and caused high fatality rate due to loss of walking ability [1–3]. In order to reduce mortality and disability rate, the general consensus in the literature is that the primary goal of intertrochanteric fracture treatment should be to obtain a stable fixation of the fracture that will allow early mobilization, restoring the function of the limb [4–6]. Early surgical procedure was crucial for the good functional outcome and for the avoidance of serious postoperative complications for the implant or the patient [5]. However, the best perioperative care for intertrochanteric femoral fracture remains controversial.

Intramedullary fixation system has mechanical and biological advantages in intertrochanteric fracture [1–5]. PFNA is an improvement based on AO/ASIF PFN which is designed to overcome some of the difficulties encountered with earlier designs of intramedullary proximal femoral nails. The inserted PFNA blade achieves an excellent fit through cancellous bone compaction and requires less bone removal compared to a traditional lag screw. These characteristics of PFNA provide optimal anchoring and stability which have been bio-mechanically proven to retard rotation and varus collapse [6–9]. Therefore, PFNA is more suitable for the elderly particularly combined with severe osteoporosis patients. However, the latent blood loss caused by trauma and surgery still cannot be ignored despite the above advantages which may result in prolonged LOS and poor postoperative rehabilitation effect.

A long LOS for intertrochanteric fracture patients is not desirable, as they have an increased risk of developing complications and losing their independence [12, 13]. Furthermore, decreasing LOS and readmission rates have become a major focus of cost reduction in intertrochanteric fracture surgery [15]. The decrease in LOS achieved with ERAS pathway results in a corresponding decrease in complications have been reported in arthroplasty domain [18]. Additionally, a large national cohort demonstrated that the reduction in LOS did not coincide with an increase in readmissions which was encouraging [21]. Our results also showed a decrease in LOS in trauma patients. Intertrochanteric fracture patients who have received ERAS pathway care have better mobilization subsequently, more likely to discharge home earlier rather than require longer nursing care in hospital, and therefore result in a corresponding decrease in cost. Taken together, the global costs of intertrochanteric fracture therapy can be substantially reduced with the combination of decreased LOS, decreased readmissions, and a decrease in complication rates.

Several clinical studies involving ERAS on arthroplasty have been described previously in the literature [18–20], but few have had this extent of impact on trauma surgical procedures. This article has provided a new perspective of ERAS pathway in intertrochanteric fracture surgical procedures. The concept of ERAS rehabilitation is now established as safe and effective. Patients treated according to ERAS principles can expect a faster recovery without increased adverse events. Other benefits of the ERAS approach include a reduction in complications, early mobilization, pain, and LOS. However, despite the clear benefits of ERAS care, implementation in daily practice has been slow. Several cross-sectional surveys have documented that perioperative care is still traditional in many institutions [14–18]. ERAS surgery requires organizational changes within the hospital, and therefore is difficult to achieve. The translation of ERAS methodology into routine clinical practice is dependent on multidisciplinary collaborations, routine training and education among doctors and nurses, and identification of cultural barriers, etc. [16–19].

In the ERAS concept, pain management is one of the most important constituents. Postoperative pain not only prolongs the LOS, but also reduces the subjective willingness of patients to take early rehabilitation exercises, and therefore resulting in decreased joint function [17, 18]. The traditional procedure is to give appropriate measures when the patient complains unbearable pain. As for the treatment of postoperative pain, the idea of ERAS was to give COX-2 inhibitor analgesia before the complete extinction of anesthesia. If the patient’s VAS score was still greater than 4, we should take pain management and additional opioids could be added. The advantage of applying selective COX-2 inhibitors included less gastrointestinal response, reducing the number of opioids and reducing opioid-related over-sedation [21]. In this study, advanced analgesia and multi-mode analgesia were used in the ERAS group. The VAS score was statistically significant compared with the traditional rehabilitation group. It is worth noting that the patient’s stress response to trauma had already occurred before admission. It is due to the emphasis of the ERAS concept on pain management and the application of advanced analgesia that these adverse factors have reduced the influence on the body’s stress response and therefore promoted the rapid rehabilitation.

For elderly patients, early rehabilitation exercise is very important, ERAS concept emphasizes early functional rehabilitation which increases intestinal peristalsis and lung capacity, promotes lower limb muscle strength recovery, maintains hip activity, accelerates the blood circulation at the incision site, promotes incision healing and lower limb venous reflux, and prevents the formation of deep venous thrombosis after operation [13–17]. Although the local nerve block anesthesia using the small dosage and lower concentration can achieve the maximal analgesic effect, the lower limb muscle strength may be affected after the femoral nerve block, and therefore delay the early mobilization [19]. Therefore, the two groups of patients in this study did not use local nerve block analgesia. The decreasing risk of lower limb thrombosis and muscle atrophy demonstrated by the improved HHS and ADL scores in the ERAS group was particularly useful for the recovery of hip function especially during perioperative period.

The limitations of this study are primarily the result of its retrospective design. In addition, the number of cases is small and the follow-up period is somewhat short. Nevertheless, the intraoperative and postoperative protocols were uniform. Besides, we have not reported on more detailed functional outcomes such as walking distance, ability to walk independently and climb stairs, return to work and driving after the discharge or blood loss during the procedure, which is also a limitation of our existing ERAS database. The results of this study need to be substantiated with adequately powered randomized control trials.

## Conclusion

In conclusion, an orthopedic-specific ERAS program implementation in intertrochanteric fracture independently reduces median LOS without compromising early functional outcome. The improvement methodology not only supports the implementation of ERAS protocols but also establishes change that is sustainable with minimal additional resource use. This has not previously been reported. Further studies should be undertaken to determine the relative importance of various individual measures and the longer-term functional outcome in patients treated on ERAS.

## Data Availability

The datasets used and/or analyzed during the current study are available from the corresponding author on reasonable request.

## Abbreviations

ADL: Activity of daily living scale
AF: Atrial fibrillation
ASIF: Association for the Study of Internal Fixation
BMI: Body mass index
COPD: Chronic obstructive pulmonary disease
CVA: Cerebral Vascular Accident
DVT: Deep venous thrombosis
ERAS: Enhanced recovery after surgery
GI: Gastrointestinal
HHS: Harris hip scores
IHD: Ischemic heart disease
LOS: Hospital length of stay
MI: Myocardial infarction
PE: Pulmonary embolism
PFN: Proximal femoral nail
PFNA: Proximal femoral nail anti-rotation
POD: Postoperative day
RTI: Respiratory tract infection
UTI: Urinary tract infection
VAS: Visual analog scale
